# Cognitive Psychology: First language and false memories

**DOI:** 10.1038/s44271-023-00015-y

**Published:** 2023-09-11

**Authors:** Marike Schiffer

**Affiliations:** Communications Psychology, https://www.nature.com/commspsychol/

**Keywords:** Human behaviour, Learning and memory, Cognitive neuroscience

## Abstract

When bilinguals perform a memory task in their second rather than their first language they are less likely to confuse lures for real memories or to agree with false information shared by another eye-witness, reports a study in *Journal of Experimental Psychology: General*.

**Figure:**
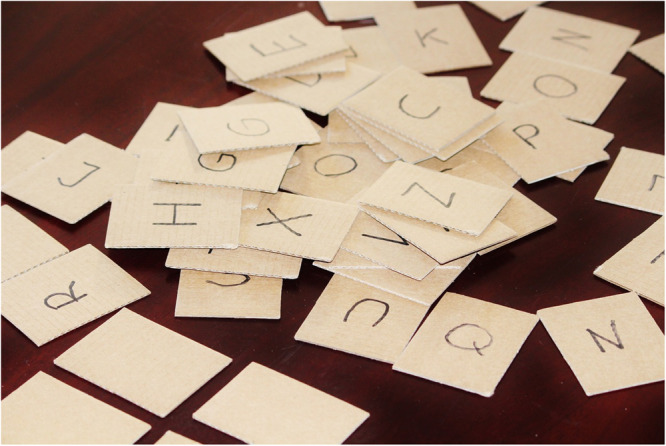
Ulrike Mai from Pixabay

We generally have the impression that our memories are a reliable representation of things that we experienced in the past. But research, for example in the context of witness testimony, has shown that our memories can be malleable; memories that we are confident are true may not be accurate at all. If our memories are not internal picture-perfect copies of our past, that raises the question: when do we (mis)remember?

Leigh Grant and colleagues at the University of Chicago reasoned that since the language can affect how we think, what language we use may also affect whether we misremember something^1^. The interesting idea the authors explored was whether participants’ would be more susceptible to recalling false memories in their first or second language. The authors hypothesized that using a second language may suppress quick, intuitive responding and thus reduce the number of false memories.

Across two studies, the researchers found that participants were less likely to fall for misleading information in their reported memory when they responded in their second language: they were more likely to catch items that were similar to previously presented stimuli but had not been encountered and were less likely to agree with false information shared by another eye-witness of a previously witnessed event.

The study raises a host of new questions. Testing for the replication of the effect in more applied settings will be important to understand the implications of the work.
